# Supplementary material for: Comparative genomics provides new insights into the diversity, physiology, and sexuality of the only industrially exploited tremellomycete: Phaffia rhodozyma
Source: BMC Genomics. 2016 Nov 9;17:901. doi: 10.1186/s12864-016-3244-7 (PMC5103461; doi:10.1186/s12864-016-3244-7)
Supplement: Additional file 6: — List of orphan genes with links to PFAM (related to Additional file 1: Table S1). (ZIP 1428 kb) [file 12864_2016_3244_MOESM6_ESM.zip › BLAST_HTML_FTR/G02428_P.html]

BLAST Search Results


```
BLASTP 2.2.27+


Reference:
Stephen F. Altschul, Thomas L. Madden, Alejandro A. Schäffer,
Jinghui Zhang, Zheng Zhang, Webb Miller, and David J. Lipman (1997),
"Gapped BLAST and PSI-BLAST: a new generation of protein database
search programs", Nucleic Acids Res. 25:3389-3402.


Reference for
composition-based statistics:
Alejandro A. Schäffer, L. Aravind, Thomas L. Madden, Sergei
Shavirin, John L. Spouge, Yuri I. Wolf, Eugene V. Koonin, and
Stephen F. Altschul (2001), "Improving the accuracy of PSI-BLAST
protein database searches with composition-based statistics and
other refinements", Nucleic Acids Res. 29:2994-3005.


Database: nr
           71,551,133 sequences; 26,053,659,533 total letters


Query= G02428_P

Length=226
                                                                      Score     E
Sequences producing significant alignments:                          (Bits)  Value

emb|CED84669.1|  hypothetical protein [Xanthophyllomyces dendrorh...   460    2e-162
gb|KMZ72444.1|  putative RNA-binding protein with multiple splici...  40.4    0.83  
ref|XP_011079763.1|  PREDICTED: uncharacterized protein LOC105163...  39.7    2.0   
ref|XP_011079764.1|  PREDICTED: uncharacterized protein LOC105163...  39.7    2.1   
ref|XP_011079762.1|  PREDICTED: uncharacterized protein LOC105163...  39.7    2.2   
ref|XP_010248620.1|  PREDICTED: uncharacterized protein LOC104591...  38.5    4.0   
ref|XP_012832781.1|  PREDICTED: uncharacterized protein LOC105953...  38.5    5.2   
ref|XP_012689005.1|  PREDICTED: Na(+)/H(+) exchange regulatory co...  38.1    5.2   
gb|EYU46643.1|  hypothetical protein MIMGU_mgv1a001710mg [Erythra...  38.5    5.2   
ref|XP_012832789.1|  PREDICTED: uncharacterized protein LOC105953...  38.5    5.3   
emb|CDO97764.1|  unnamed protein product [Coffea canephora]           38.1    5.5   


 >emb|CED84669.1| hypothetical protein [Xanthophyllomyces dendrorhous]
Length=225

 Score =  460 bits (1184),  Expect = 2e-162, Method: Compositional matrix adjust.
 Identities = 224/225 (99%), Positives = 224/225 (99%), Gaps = 0/225 (0%)

Query  1    MSNSFKSYVGRTSSVGTSSKPTRKTPSRKTAEKDSFKSLIFEEDIETESVDHRKKNTFEK  60
            MSNSFKSYVGRTSSVGTSSKPTRKTPSRKTAEKDSFKSLIFEEDIETESVDHRKKNTFEK
Sbjct  1    MSNSFKSYVGRTSSVGTSSKPTRKTPSRKTAEKDSFKSLIFEEDIETESVDHRKKNTFEK  60

Query  61   RKRKRREDAVVEEPDSFDESDKQMETDEEQGHTGEISEGVAGSQIDLVRLSEEFHARRAN  120
            RKRKRREDAVVEEPDSFDESDKQMETDEEQGHTGEISEGVAGSQIDLVRLSEEF ARRAN
Sbjct  61   RKRKRREDAVVEEPDSFDESDKQMETDEEQGHTGEISEGVAGSQIDLVRLSEEFRARRAN  120

Query  121  KSAVLRDSYTTKVVAPIINARQSVLSQWEKMDAQMAERMDCLRMTLAPTQLDVSIYSSLG  180
            KSAVLRDSYTTKVVAPIINARQSVLSQWEKMDAQMAERMDCLRMTLAPTQLDVSIYSSLG
Sbjct  121  KSAVLRDSYTTKVVAPIINARQSVLSQWEKMDAQMAERMDCLRMTLAPTQLDVSIYSSLG  180

Query  181  AQRELASQTLCSAYERCTRQLVPEVLGLADDDLNSCKPTISPVLI  225
            AQRELASQTLCSAYERCTRQLVPEVLGLADDDLNSCKPTISPVLI
Sbjct  181  AQRELASQTLCSAYERCTRQLVPEVLGLADDDLNSCKPTISPVLI  225


>gb|KMZ72444.1| putative RNA-binding protein with multiple splicing [Zostera 
marina]
Length=321

 Score = 40.4 bits (93),  Expect = 0.83, Method: Compositional matrix adjust.
 Identities = 28/73 (38%), Positives = 39/73 (53%), Gaps = 13/73 (18%)

Query  25   TPSRKTAEKDSFKSLIFEEDIETESVDH---RKKNTFEKRKRKRREDAVVEEPDSFDESD  81
            +P    A KDS + L+F+ D    SV H    KKN F KR        VV +P++FD+S 
Sbjct  83   SPHFSIAAKDSLQGLVFDPD--KNSVLHAEMAKKNLFIKR-------GVVTDPNTFDQS-  132

Query  82   KQMETDEEQGHTG  94
            K++ T  +  HTG
Sbjct  133  KRLRTGGDYTHTG  145


>ref|XP_011079763.1| PREDICTED: uncharacterized protein LOC105163202 isoform X2 [Sesamum 
indicum]
Length=896

 Score = 39.7 bits (91),  Expect = 2.0, Method: Compositional matrix adjust.
 Identities = 28/93 (30%), Positives = 44/93 (47%), Gaps = 11/93 (12%)

Query  16   GTSSKPTRKTPSRKTAEKDSFKSLIFEEDIETESVDHRKKNTFEKRKRKRR---------  66
            G+ S PTR   +    + D  K  + EE +E E VD    +  ++RKRKR          
Sbjct  625  GSDSSPTRGKNTITRVDVDHIKGSVLEETLEDEKVDAIHSDEKQQRKRKRTIMNDKQIAL  684

Query  67   -EDAVVEEPDSF-DESDKQMETDEEQGHTGEIS  97
             E A+V+EPD   + +  +M  D+   H  E++
Sbjct  685  IESALVDEPDMHRNSTSLRMWADKLSLHGAEVT  717


>ref|XP_011079764.1| PREDICTED: uncharacterized protein LOC105163202 isoform X3 [Sesamum 
indicum]
Length=822

 Score = 39.7 bits (91),  Expect = 2.1, Method: Compositional matrix adjust.
 Identities = 28/93 (30%), Positives = 44/93 (47%), Gaps = 11/93 (12%)

Query  16   GTSSKPTRKTPSRKTAEKDSFKSLIFEEDIETESVDHRKKNTFEKRKRKRR---------  66
            G+ S PTR   +    + D  K  + EE +E E VD    +  ++RKRKR          
Sbjct  551  GSDSSPTRGKNTITRVDVDHIKGSVLEETLEDEKVDAIHSDEKQQRKRKRTIMNDKQIAL  610

Query  67   -EDAVVEEPDSF-DESDKQMETDEEQGHTGEIS  97
             E A+V+EPD   + +  +M  D+   H  E++
Sbjct  611  IESALVDEPDMHRNSTSLRMWADKLSLHGAEVT  643


>ref|XP_011079762.1| PREDICTED: uncharacterized protein LOC105163202 isoform X1 [Sesamum 
indicum]
Length=906

 Score = 39.7 bits (91),  Expect = 2.2, Method: Compositional matrix adjust.
 Identities = 28/93 (30%), Positives = 44/93 (47%), Gaps = 11/93 (12%)

Query  16   GTSSKPTRKTPSRKTAEKDSFKSLIFEEDIETESVDHRKKNTFEKRKRKRR---------  66
            G+ S PTR   +    + D  K  + EE +E E VD    +  ++RKRKR          
Sbjct  635  GSDSSPTRGKNTITRVDVDHIKGSVLEETLEDEKVDAIHSDEKQQRKRKRTIMNDKQIAL  694

Query  67   -EDAVVEEPDSF-DESDKQMETDEEQGHTGEIS  97
             E A+V+EPD   + +  +M  D+   H  E++
Sbjct  695  IESALVDEPDMHRNSTSLRMWADKLSLHGAEVT  727


>ref|XP_010248620.1| PREDICTED: uncharacterized protein LOC104591473 [Nelumbo nucifera]
Length=343

 Score = 38.5 bits (88),  Expect = 4.0, Method: Compositional matrix adjust.
 Identities = 31/115 (27%), Positives = 57/115 (50%), Gaps = 18/115 (16%)

Query  117  RRANKSAVLRDSYTTKVVAPIINARQSVLSQWEKMDAQMAERMDCLRMTL----APTQLD  172
            R+  + A+    +   +VA +I  R++ +      DAQ+AE ++ +   +     P  +D
Sbjct  198  RKYIRYAMNEKPFNADLVADLIQLRKASMLD----DAQVAEILNEISRRIVREKGPVVMD  253

Query  173  VSIYSSLGAQRELASQTLCSAY-------ERCTRQ---LVPEVLGLADDDLNSCK  217
            +S YS  G +R+LA Q L           E C+R    +V E+ G+ D+DL++ +
Sbjct  254  MSGYSEKGLKRKLAVQALFGKIFYLSELPEFCSRDSSLIVKEIFGVTDEDLDTLR  308


>ref|XP_012832781.1| PREDICTED: uncharacterized protein LOC105953650 isoform X1 [Erythranthe 
guttatus]
Length=900

 Score = 38.5 bits (88),  Expect = 5.2, Method: Compositional matrix adjust.
 Identities = 26/70 (37%), Positives = 33/70 (47%), Gaps = 10/70 (14%)

Query  16   GTSSKPTRKTPSRKTAEKDSFKSLIFEEDIETESVDHRKKNTFEKRKRKRR---------  66
            GT S PTR   S    + D  K   FEE +E E  D    +  ++RKRKR          
Sbjct  624  GTDSSPTRGKNSSDLMDVDHVKGSGFEETMEDEKADAMYSDEKQQRKRKRTIMNDRQIAL  683

Query  67   -EDAVVEEPD  75
             E A+V+EPD
Sbjct  684  IESALVDEPD  693


>ref|XP_012689005.1| PREDICTED: Na(+)/H(+) exchange regulatory cofactor NHE-RF2-like 
[Clupea harengus]
Length=247

 Score = 38.1 bits (87),  Expect = 5.2, Method: Compositional matrix adjust.
 Identities = 38/117 (32%), Positives = 58/117 (50%), Gaps = 26/117 (22%)

Query  19   SKPTRKTPSRKTAEKDSFKSLIFEEDIETESVDHRKKNTFEKRKRKRREDAVVEEPDSFD  78
            SKPTR++PSR   + +          +  E++ H +   F K    R    +V +PD+ D
Sbjct  130  SKPTRRSPSRAVKKVNG---------VNIENMRHAEVVAFIKSG-GRETRLLVVDPDT-D  178

Query  79   ESDKQMETDEEQGHTGEISEGVAGSQIDL-----VRLS-------EEFHARRANKSA  123
            E  K+M      GH   +SEG +GS++D      +RLS       E+ HA+RA+K A
Sbjct  179  EHLKRMGVTPTSGH---MSEGSSGSRLDPFAEMGLRLSQTAAEAREKAHAKRAHKRA  232


>gb|EYU46643.1| hypothetical protein MIMGU_mgv1a001710mg [Erythranthe guttata]
Length=770

 Score = 38.5 bits (88),  Expect = 5.2, Method: Compositional matrix adjust.
 Identities = 26/70 (37%), Positives = 33/70 (47%), Gaps = 10/70 (14%)

Query  16   GTSSKPTRKTPSRKTAEKDSFKSLIFEEDIETESVDHRKKNTFEKRKRKRR---------  66
            GT S PTR   S    + D  K   FEE +E E  D    +  ++RKRKR          
Sbjct  540  GTDSSPTRGKNSSDLMDVDHVKGSGFEETMEDEKADAMYSDEKQQRKRKRTIMNDRQIAL  599

Query  67   -EDAVVEEPD  75
             E A+V+EPD
Sbjct  600  IESALVDEPD  609


>ref|XP_012832789.1| PREDICTED: uncharacterized protein LOC105953650 isoform X2 [Erythranthe 
guttatus]
Length=899

 Score = 38.5 bits (88),  Expect = 5.3, Method: Compositional matrix adjust.
 Identities = 26/70 (37%), Positives = 33/70 (47%), Gaps = 10/70 (14%)

Query  16   GTSSKPTRKTPSRKTAEKDSFKSLIFEEDIETESVDHRKKNTFEKRKRKRR---------  66
            GT S PTR   S    + D  K   FEE +E E  D    +  ++RKRKR          
Sbjct  623  GTDSSPTRGKNSSDLMDVDHVKGSGFEETMEDEKADAMYSDEKQQRKRKRTIMNDRQIAL  682

Query  67   -EDAVVEEPD  75
             E A+V+EPD
Sbjct  683  IESALVDEPD  692


>emb|CDO97764.1| unnamed protein product [Coffea canephora]
Length=328

 Score = 38.1 bits (87),  Expect = 5.5, Method: Compositional matrix adjust.
 Identities = 25/73 (34%), Positives = 40/73 (55%), Gaps = 13/73 (18%)

Query  25   TPSRKTAEKDSFKSLIFEEDIETESVDH---RKKNTFEKRKRKRREDAVVEEPDSFDESD  81
            TP +  A KD+ + L+F  D E+ S+ H    KKN F KR        +V + +++D+S 
Sbjct  100  TPQQAIAAKDALRDLVF--DAESNSILHTEMAKKNLFVKR-------GIVADSNAYDQS-  149

Query  82   KQMETDEEQGHTG  94
            K++ T  +  HTG
Sbjct  150  KRLRTGGDYTHTG  162


Lambda      K        H        a         alpha
   0.314    0.127    0.349    0.792     4.96 

Gapped
Lambda      K        H        a         alpha    sigma
   0.267   0.0410    0.140     1.90     42.6     43.6 

Effective search space used: 1269978365088


  Database: nr
    Posted date:  Sep 23, 2015 12:05 AM
  Number of letters in database: 26,053,659,533
  Number of sequences in database:  71,551,133


Matrix: BLOSUM62
Gap Penalties: Existence: 11, Extension: 1
Neighboring words threshold: 11
Window for multiple hits: 40
```
